# Combined Selenium and Iodine Application Modulates Antioxidant and Photosynthetic Responses in Raspberry Seedlings Under Heat Stress

**DOI:** 10.3390/plants15142146

**Published:** 2026-07-12

**Authors:** Carlos Henrique Milagres Ribeiro, Everton Geraldo de Morais, Pedro Antônio Namorato Benevenute, Anyela Pierina Vega Quispe, Jucelino de Sousa Lima, Paulo Eduardo Ribeiro Marchiori, Antonio Chalfun-Junior, Leônidas Canuto dos Santos, Luiz Roberto Guimarães Guilherme, Rafael Pio

**Affiliations:** 1 Department of Soil Science, Federal University of Lavras, University Campus, P.O. Box 3037, Lavras 37203-202, MG, Brazil; evertonmoraislp@gmail.com (E.G.d.M.); benevenutepedro@gmail.com (P.A.N.B.); anyela.quispe1@estudante.ufla.br (A.P.V.Q.); leonidas.santos2@estudante.ufla.br (L.C.d.S.); guilherm@ufla.br (L.R.G.G.); 2 Department of Biology, Institute of Natural Sciences, Federal University of Lavras, University Campus, P.O. Box 3037, Lavras 37203-202, MG, Brazil; sousajucelino12@gmail.com (J.d.S.L.); paulo.machiori@ufla.br (P.E.R.M.); chalfunjunior@ufla.br (A.C.-J.); 3 Department of Agriculture, Federal University of Lavras, University Campus, P.O. Box 3037, Lavras 37203-202, MG, Brazil; rafaelpio@ufla.br

**Keywords:** *Rubus idaeus*, abiotic stress, antioxidants, heat tolerance, photosynthesis

## Abstract

Heat stress limits raspberry cultivation in tropical and subtropical regions, yet strategies to mitigate its effects on seedlings are scarce. Selenium (Se) and iodine (I) individually enhance plant tolerance to abiotic stress, but whether their combined application produces complementary effects on the physiological responses of heat-stressed raspberry seedlings remains unexplored. This study addressed this gap by evaluating the physiological, biochemical, and photosynthetic responses of ‘Heritage’ raspberry seedlings to foliar applications of Se and I, alone and in combination, under high-temperature conditions. The experiment used a completely randomized design with ten treatments: one non-stressed control (0 mg L^−1^ Se and 0 mg L^−1^ I) and nine heat-stressed treatments. Among the stressed treatments, one received no selenium or iodine (0 mg L^−1^ Se and 0 mg L^−1^ I) and served as the stressed control. The remaining eight treatments received combinations of selenium (0, 25, and 50 mg L^−1^) and iodine (0, 50, and 100 mg L^−1^). One week after foliar application, seedlings were exposed to heat stress under controlled conditions of 40/18 °C (day/night) for three days. Physiological, biochemical, antioxidant, and chlorophyll fluorescence parameters were evaluated during and after the stress period. During heat stress, intermediate Se and I combinations helped maintain plant water status and photosynthetic performance, although the physiological and biochemical responses varied across treatments and sampling times. After the stress period, these treatments promoted osmotic adjustment and improved antioxidant regulation, contributing to the recovery of raspberry seedlings. Principal component analysis reinforced the association between intermediate Se and I combinations and mechanisms related to antioxidant regulation, osmotic adjustment, and photoprotection. Collectively, these findings indicate that co-application of Se and I triggers complementary physiological mechanisms of heat-stress acclimation in raspberry seedlings, suggesting a promising strategy for enhancing seedling tolerance to high-temperature stress.

## 1. Introduction

Raspberry (*Rubus idaeus* L.), a temperate-climate species in the Rosaceae family, is widely cultivated and commercialized worldwide, with growing demand for both fresh consumption and use in the food industry [1]. According to the Food and Agriculture Organization [2], the main global raspberry producers are Russia, Mexico, and Serbia [1,2,3]. Beyond its economic relevance, raspberry cultivation stands out for the high nutritional and functional value of its fruits, which contain elevated levels of anthocyanins, phenolic compounds, flavonoids, and vitamin C, all of which are associated with high antioxidant capacity and beneficial effects on human health [4,5]. In recent years, raspberry cultivation has expanded into non-traditional production areas, including subtropical and tropical regions, driven by increasing market demand and the economic value of fresh fruits and processed products [6].

Despite the economic potential of this crop, raspberry cultivation in subtropical and tropical regions remains a significant challenge, especially during the initial establishment of seedlings in the field [7]. Under these conditions, high temperatures, intense solar radiation, and low relative humidity can impair plant growth, physiological stability, and seedling survival, requiring strategies that improve physiological acclimation and metabolic adjustment during establishment [8]. Furthermore, the increasing frequency and intensity of heat waves associated with climate change have intensified the challenges for temperate fruit cultivation in warmer production regions [9]. The ‘Heritage’ cultivar has attracted interest for cultivation across different regions due to its commercial relevance and adaptability to diverse production systems. However, limitations in heat-stress tolerance during seedling establishment persist [10].

Among the main abiotic factors limiting the development of temperate fruit species in regions with greater thermal amplitude, heat stress is a major constraint [11]. Exposure to elevated temperatures induces physiological, biochemical, and structural alterations in plants, including reduced photosynthetic activity, degradation of photosynthetic pigments, disruption of cellular membranes, and excessive accumulation of reactive oxygen species (ROS), such as superoxide and hydrogen peroxide (H_2_O_2_) [12,13]. The uncontrolled increase in these ROS causes oxidative damage to proteins, lipids, and nucleic acids, compromising cellular integrity and metabolic function, and consequently plant growth and productivity [14].

In response to heat stress, plants activate several physiological and biochemical defense mechanisms, including antioxidant metabolism, osmotic adjustment, and photoprotective processes associated with the photosynthetic apparatus [15]. In this context, the application of beneficial elements has been considered a promising strategy to mitigate the effects of abiotic stress in fruit and vegetable crops [16]. Among these elements, selenium (Se), although not considered essential for plants, has been associated with modulation of redox homeostasis and activation of antioxidant enzymes such as superoxide dismutase (SOD) and catalase (CAT), thereby contributing to membrane stabilization and protection of the photosynthetic apparatus under stressful conditions [17]. Selenium may also help maintain electron transport stability and reduce oxidative propagation damage caused by excessive ROS accumulation [16].

Iodine (I), like selenium (Se), is not considered a plant nutrient. However, it is recognized as a functional element that participates in antioxidant regulation and physiological signaling in plants. Recent studies have demonstrated that iodine may reduce ROS accumulation, maintain cellular integrity, modulate photosynthetic activity, and stimulate stress-tolerance mechanisms [18]. In addition, iodine has been associated with maintaining photosystem II stability and regulating physiological processes involved in plant acclimation to abiotic stress [19]. Evidence suggests that selenium and iodine may exert complementary physiological functions in plant metabolism, with Se primarily associated with antioxidant defense and cellular redox regulation. In contrast, I is associated with photoprotection, signaling pathways, and reactive oxygen species detoxification [16,17,18]. Therefore, the combined application of these elements may promote complementary, concentration-dependent physiological responses associated with plant acclimation to abiotic stress, particularly under heat stress [17,18].

Although selenium and iodine have been associated with tolerance to various abiotic stresses, including salinity, drought, and oxidative stress, growing evidence indicates that both elements also contribute to plant thermotolerance by improving antioxidant regulation, preserving membrane integrity, and maintaining photosynthetic performance under high-temperature conditions [17,18,19,20,21,22]. Recent studies indicate that selenium contributes to heat-stress acclimation through antioxidant regulation, membrane stabilization, and protection of photosynthetic processes under elevated temperatures [17,23,24]. Likewise, iodine has been recognized as a functional element that modulates oxidative metabolism and maintains plant physiological performance under heat stress [18,22]. Together, these findings suggest that Se and I may support complementary physiological adjustments associated with plant acclimation to heat stress.

Although the isolated effects of Se and I have been reported in various plant species, studies examining their combined effects on raspberry plants under heat stress remain scarce, particularly under cultivation conditions in tropical and subtropical regions. While physiological, biochemical, and photochemical markers are widely used to characterize plant responses to abiotic stress, these parameters represent indirect indicators of stress acclimation and do not necessarily reflect whole-plant performance, survival, or long-term recovery under adverse conditions. Therefore, it was hypothesized that the combined application of Se and I could improve physiological responses to heat stress acclimation, including antioxidant responses, osmotic adjustment, and maintenance of photochemical performance in raspberry seedlings. Thus, the present study aimed to investigate the physiological, biochemical, and photochemical responses to the application of selenium and iodine, individually and in combination, and their association with heat-stress acclimation in raspberry seedlings of the ‘Heritage’ cultivar.

## 2. Results

**Figure 1:**
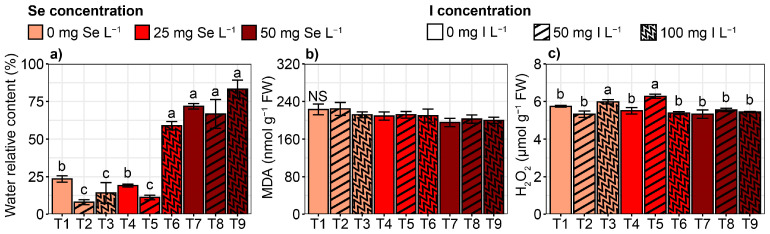
Effect of foliar application of selenium (Se) and iodine (I) applied individually or in combination on (**a**) relative water content (RWC), (**b**) malondialdehyde (MDA), and (**c**) hydrogen peroxide (H_2_O_2_) in raspberry seedlings cv. ‘Heritage’ under heat stress. The treatments included nine water-stressed treatments. Among the stressed treatments, T1 (0 mg L^−1^ Se and 0 mg L^−1^ I) served as the stressed control, while T2 (0/50), T3 (0/100), T4 (25/0), T5 (25/50), T6 (25/100), T7 (50/0), T8 (50/50), and T9 (50/100) received different combinations of selenium and iodine. Values represent Se and I concentrations (mg L^−1^), respectively. Data are presented as the mean ± standard error. Different lowercase letters indicate significant differences according to the Scott–Knott test (*p* < 0.05).

Heat stress significantly affected relative water content (RWC) and H_2_O_2_ accumulation in raspberry seedlings, whereas no significant differences were observed in malondialdehyde (MDA) content among treatments (Figure 1). Seedlings treated with combined Se and I applications containing 100 mg L^−1^ I showed higher RWC values, regardless of Se concentration. Treatments combining 25 or 50 mg L^−1^ Se with 100 mg L^−1^ I increased RWC by approximately 30% compared with the stressed control treatment (0 mg L^−1^ Se + 0 mg L^−1^ I) (Figure 1a). No significant differences were observed in MDA levels among treatments (Figure 1b). Regarding H_2_O_2_ accumulation, the application of 100 mg L^−1^ I alone and the combined treatment containing 25 mg L^−1^ Se + 50 mg L^−1^ I resulted in significantly higher values than the other treatments (Figure 1c).

**Figure 2:**
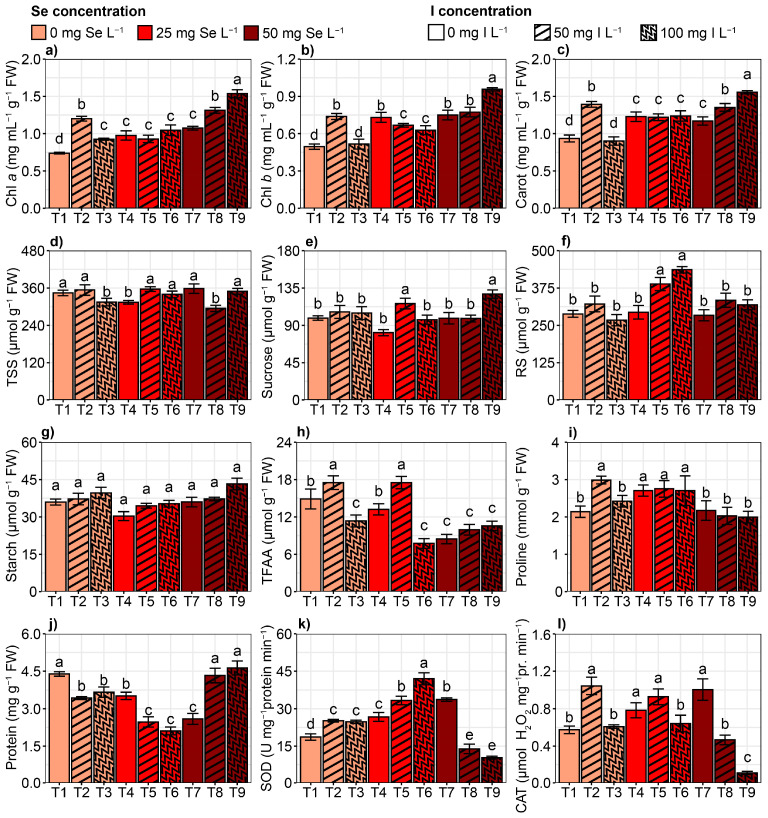
Effect of foliar application of Se and I, applied individually or in combination at different concentrations, on (**a**): chlorophyll *a*, (**b**): chlorophyll *b*, (**c**): carotenoids (Carot), (**d**): total soluble sugars (TSS), (**e**): sucrose, (**f**): reducing sugars (RS), (**g**): starch, (**h**): total free amino acids (TFAA), (**i**): proline, (**j**): proteins, and antioxidant enzyme activities of (**k**): superoxide dismutase (SOD) and (**l**): catalase (CAT) in raspberry seedlings cv. ‘Heritage’ under heat stress. CAT activity is expressed as µmol H_2_O_2_ mg^−1^ protein min^−1^. The treatments included nine treatments under heat stress. Among them, T1 (0 mg L^−1^ Se and 0 mg L^−1^ I) served as the stress control, while treatments T2 (0/50), T3 (0/100), T4 (25/0), T5 (25/50), T6 (25/100), T7 (50/0), T8 (50/50), and T9 (50/100) received different combinations of selenium and iodine. Values represent Se and I concentrations (mg L^−1^), respectively. Data are presented as the mean ± standard error. Different lowercase letters indicate significant differences among treatments according to the Scott–Knott test (*p* < 0.05).

Heat stress significantly affected the accumulation of photosynthetic pigments, osmolytes, carbohydrates, proteins, and antioxidant enzyme activity in raspberry seedlings treated with Se and I (Figure 2). Higher chlorophyll *a* (Chl *a*) (Figure 2a), chlorophyll *b* (Chl *b*) (Figure 2b), and carotenoid contents (Figure 2c) were observed in seedlings treated with the combined application of 50 mg L^−1^ Se + 100 mg L^−1^ I. Compared with the stressed control (0 mg L^−1^ Se + 0 mg L^−1^ I), these treatments increased Chl *a*, Chl *b*, and carotenoid contents by approximately 70%, 80%, and 65%, respectively. For total soluble sugars (TSS) (Figure 2d), higher values were observed in seedlings treated with 0 mg L^−1^ Se + 50 mg L^−1^ I, 25 mg L^−1^ Se combined with 50 or 100 mg L^−1^ I, 50 mg L^−1^ Se alone, and 50 mg L^−1^ Se + 100 mg L^−1^ I. In contrast, lower values were recorded in the remaining treatments. Sucrose content (Figure 2e) was higher in seedlings treated with 25 mg L^−1^ Se + 50 mg L^−1^ I, while reducing sugars were significantly increased in treatments containing 25 mg L^−1^ Se combined with 50 or 100 mg L^−1^ I. No significant differences were observed in starch content among treatments.

Higher total free amino acid contents (TFAA) were observed in seedlings treated with 0 mg L^−1^ Se + 50 mg L^−1^ I and 25 mg L^−1^ Se + 50 mg L^−1^ I. In contrast, lower values were recorded in treatments containing 100 mg L^−1^ I, which were associated with higher Se concentrations (Figure 2f). Proline accumulation (Figure 2i) was greater in seedlings treated with 0 mg L^−1^ Se + 50 mg L^−1^ I and in treatments containing 25 mg L^−1^ Se, regardless of iodine concentration. Soluble protein content was higher in the stressed control and in seedlings treated with 50 mg L^−1^ Se combined with 50 or 100 mg L^−1^ I, whereas lower values were observed in treatments containing 25 mg L^−1^ Se combined with iodine (Figure 2j). Regarding antioxidant enzymes, superoxide dismutase (SOD) activity (Figure 2k) was highest in seedlings treated with 25 mg L^−1^ Se + 100 mg L^−1^ I. By contrast, lower values were observed in treatments containing 50 mg L^−1^ Se combined with iodine. Catalase (CAT) activity was higher in seedlings treated with 0 mg L^−1^ Se + 50 mg L^−1^ I, 25 mg L^−1^ Se alone, 25 mg L^−1^ Se + 50 mg L^−1^ I, and 50 mg L^−1^ Se alone, whereas the lowest CAT activity was observed in seedlings treated with 50 mg L^−1^ Se + 100 mg L^−1^ I (Figure 2l).

**Figure 3:**
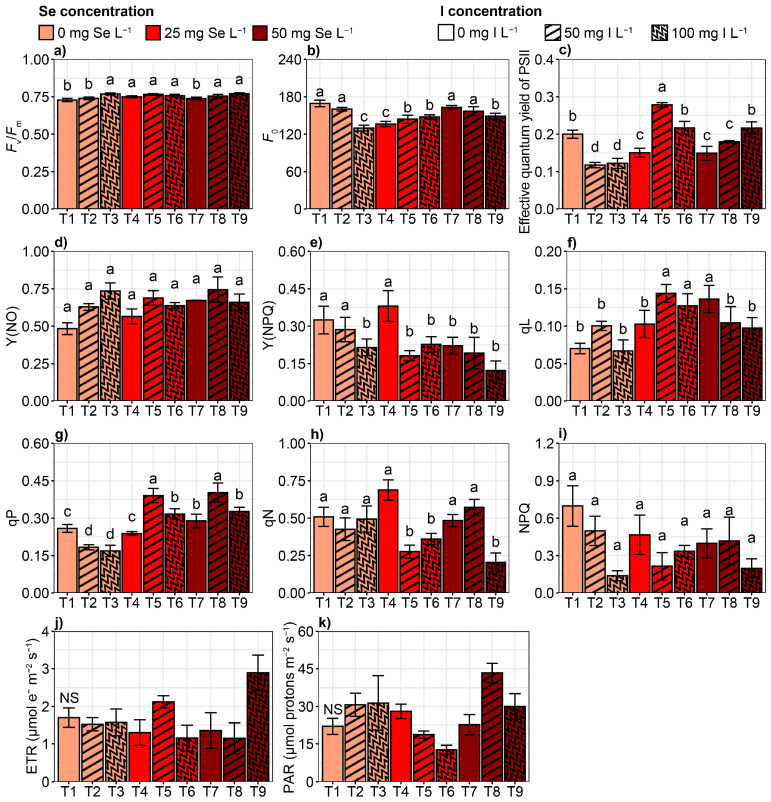
Effect of foliar application of Se and I, applied individually or in combination at different concentrations, on chlorophyll fluorescence parameters obtained using MINI-PAM in raspberry seedlings cv. ‘Heritage’ under heat stress. (**a**): Maximum quantum efficiency of PSII (*F*_v_/*F*_m_), (**b**): minimum fluorescence (*F*_0_), (**c**): effective quantum yield of PSII [Y(II)], (**d**): quantum yield of non-regulated energy dissipation [Y(NO)], (**e**): quantum yield of regulated energy dissipation [Y(NPQ)], (**f**): photochemical quenching coefficient based on the lake model (qL), (**g**): photochemical quenching coefficient (qP), (**h**): non-photochemical quenching coefficient (qN), (**i**): non-photochemical quenching (NPQ), (**j**): electron transport rate (ETR), and (**k**): photosynthetically active radiation (PAR). The treatments included nine treatments under heat stress. Among them, T1 (0 mg L^−1^ Se and 0 mg L^−1^ I) served as the stress control, while treatments T2 (0/50), T3 (0/100), T4 (25/0), T5 (25/50), T6 (25/100), T7 (50/0), T8 (50/50), and T9 (50/100) received different combinations of selenium and iodine. Data are presented as the mean ± standard error. Different lowercase letters indicate significant differences among treatments according to the Scott–Knott test (*p* < 0.05).

Heat stress significantly affected chlorophyll fluorescence parameters in raspberry seedlings treated with Se and I (Figure 3). Higher maximum quantum efficiency of photosystem II (*F*_v_/*F*_m_) values (Figure 3a) were observed in seedlings treated with 100 mg L^−1^ I alone; in treatments containing 25 mg L^−1^ Se, regardless of iodine concentration; and in the combination of 50 mg L^−1^ Se + 100 mg L^−1^ I. Lower values were recorded in the stressed control, 0 mg L^−1^ Se + 50 mg L^−1^ I, and 50 mg L^−1^ Se alone. For *F*_0_ (Figure 3b), higher values were observed in the stressed control and in treatments containing 50 mg L^−1^ Se, whereas lower values were recorded in seedlings treated with 100 mg L^−1^ I alone and with 25 mg L^−1^ Se alone. The effective quantum yield of PSII (Figure 3c) was highest in seedlings treated with 25 mg L^−1^ Se + 50 mg L^−1^ I, while lower values were observed in treatments containing iodine alone (50 and 100 mg L^−1^ I). No significant differences were observed among treatments for Y(NO) (Figure 3d), ETR (Figure 3j), and PAR (Figure 3k). Higher qL values (Figure 3f) were observed in seedlings treated with 25 mg L^−1^ Se combined with 50 or 100 mg L^−1^ I, and in 50 mg L^−1^ Se alone, whereas lower values were recorded in the stressed control and in seedlings treated with iodine alone. Similarly, qP values were higher in treatments containing 25 mg L^−1^ Se + 50 mg L^−1^ I and 50 mg L^−1^ Se + 50 mg L^−1^ I (Figure 3g).

For qN, higher values were observed in the stressed control, iodine-only treatments, 25 mg L^−1^ Se alone, and 50 mg L^−1^ Se combined with 50 mg L^−1^ I. In contrast, lower values were recorded in treatments combining 25 mg L^−1^ Se with iodine, and in 50 mg L^−1^ Se + 100 mg L^−1^ I (Figure 3h). Regarding NPQ, higher values were observed in the stressed control, 0 mg L^−1^ Se + 50 mg L^−1^ I, and 25 mg L^−1^ Se alone, whereas the remaining treatments showed lower, statistically similar values (Figure 3i).

**Figure 4:**
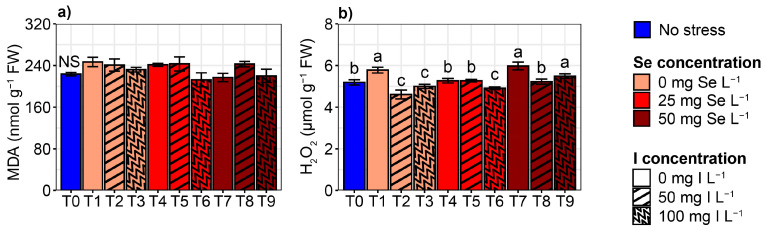
Effect of foliar application of Se and I, applied individually or in combination at different concentrations, on (**a**) malondialdehyde (MDA) and (**b**) hydrogen peroxide (H_2_O_2_) contents in raspberry seedlings cv. ‘Heritage’ under heat stress, including an additional non-stressed control treatment used as a physiological reference. The treatments included nine treatments under heat stress. Among them, T1 (0 mg L^−1^ Se and 0 mg L^−1^ I) served as the stress control, while treatments T2 (0/50), T3 (0/100), T4 (25/0), T5 (25/50), T6 (25/100), T7 (50/0), T8 (50/50), and T9 (50/100) received different combinations of selenium and iodine. Data are presented as the mean ± standard error. Different lowercase letters indicate significant differences among treatments according to the Scott–Knott test (*p* < 0.05).

After heat stress exposure, no significant differences in MDA content were observed among treatments, indicating similar lipid peroxidation levels under the evaluated conditions (Figure 4a). In contrast, H_2_O_2_ accumulation varied significantly among treatments. Higher H_2_O_2_ levels were observed in the stressed control, in seedlings treated with 50 mg L^−1^ Se alone, and in the combined treatment containing 50 mg L^−1^ Se + 100 mg L^−1^ I. Lower H_2_O_2_ concentrations were recorded in seedlings treated with iodine alone (50 and 100 mg L^−1^ I) and in the combination of 25 mg L^−1^ Se + 100 mg L^−1^ I (Figure 4b).

**Figure 5:**
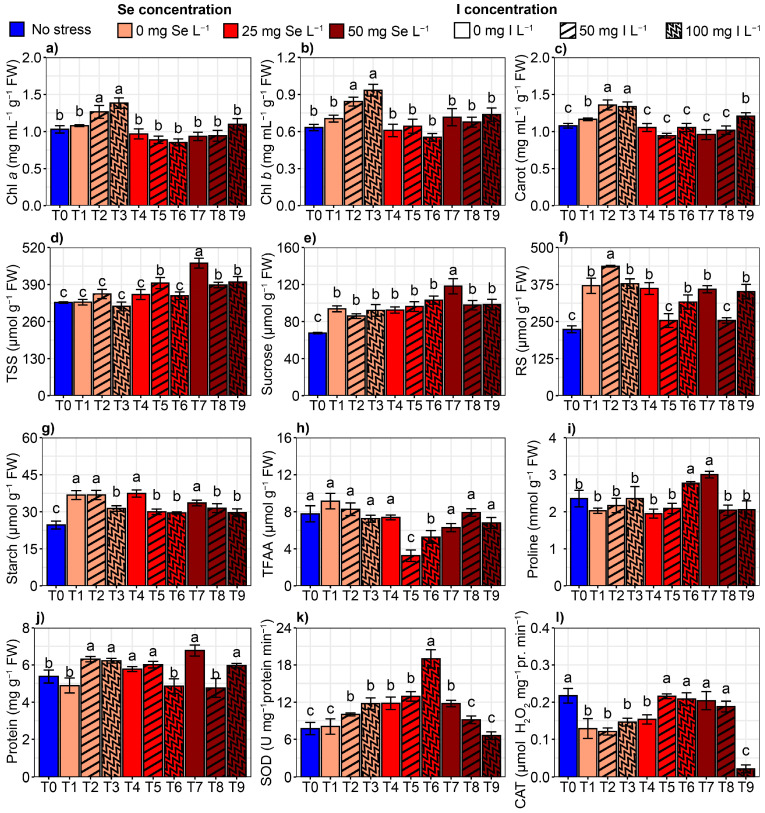
Effect of foliar application of Se and I, applied individually or in combination at different concentrations, on (**a**): chlorophyll *a*, (**b**): chlorophyll *b*, (**c**): carotenoids (Carot), (**d**): total soluble sugars (TSS), (**e**): sucrose, (**f**): reducing sugars (RS), (**g**): starch, (**h**): total free amino acids (TFAA), (**i**): proline, (**j**): proteins, and antioxidant enzyme activities of (**k**): superoxide dismutase (SOD) and (**l**): catalase (CAT) in raspberry seedlings cv. ‘Heritage’ under non-stress and heat stress. The treatments included one non-stressed control (T0; 0 mg L^−1^ Se and 0 mg L^−1^ I) and nine stressed treatments. Among the stressed treatments, T1 (0 mg L^−1^ Se and 0 mg L^−1^ I) served as the stressed control, while T2 (0/50), T3 (0/100), T4 (25/0), T5 (25/50), T6 (25/100), T7 (50/0), T8 (50/50), and T9 (50/100) received different combinations of selenium and iodine. Values represent Se and I concentrations (mg L^−1^), respectively. Data are presented as the mean ± standard error. Different lowercase letters indicate significant differences among treatments according to the Scott–Knott test (*p* < 0.05).

Regarding photosynthetic pigments, seedlings treated with iodine alone (50 and 100 mg L^−1^ I) had higher chlorophyll *a* (Figure 5a), chlorophyll *b* (Figure 5b), and carotenoid (Figure 5c) contents than the other treatments. For total soluble sugars (TSS) (Figure 5d), the highest values were observed in seedlings treated with 50 mg L^−1^ Se alone. In contrast, lower values were recorded in the non-stressed control and in treatments with lower Se concentrations. Similarly, sucrose content was higher in seedlings treated with 50 mg L^−1^ Se alone, whereas the non-stressed control showed the lowest values (Figure 5e). Reducing sugar content was significantly higher in seedlings treated with 0 mg L^−1^ Se + 50 mg L^−1^ I, whereas lower values were observed in the non-stressed control and in treatments containing 25 mg L^−1^ Se + 50 mg L^−1^ I and 50 mg L^−1^ Se + 50 mg L^−1^ I (Figure 5f). Higher starch accumulation was observed in the stressed control, in seedlings treated with iodine alone (50 mg L^−1^ I), in 25 mg L^−1^ Se alone, and in 50 mg L^−1^ Se alone, whereas the non-stressed control showed the lowest starch content (Figure 5g).

For total free amino acids (TFAA), most treatments showed similar, elevated values, whereas the combined treatment of 25 mg L^−1^ Se + 50 mg L^−1^ I had the lowest content (Figure 5h). Proline accumulation was higher in seedlings treated with 25 mg L^−1^ Se + 100 mg L^−1^ I and with 50 mg L^−1^ Se alone than in the remaining treatments. Soluble protein content (Figure 5i) was higher in seedlings treated with iodine alone (50 and 100 mg L^−1^ I), in treatments containing 25 mg L^−1^ Se, and in the combined treatment of 50 mg L^−1^ Se + 100 mg L^−1^ I. In contrast, lower values were observed in the non-stressed control, the stressed control, and the treatments 25 mg L^−1^ Se + 100 mg L^−1^ I and 50 mg L^−1^ Se + 50 mg L^−1^ I. Regarding antioxidant enzymes, the highest SOD activity was observed in seedlings treated with 25 mg L^−1^ Se + 100 mg L^−1^ I. In contrast, lower values were recorded in the non-stressed control, the stressed control, and treatments containing 50 mg L^−1^ Se combined with iodine (Figure 5k). Higher CAT activity was observed in the non-stressed control and in seedlings treated with 25 mg L^−1^ Se + 50 or 100 mg L^−1^ I, as well as in seedlings treated with 50 mg L^−1^ Se alone or combined with 50 mg L^−1^ I. In contrast, the lowest CAT activity was recorded in seedlings treated with 50 mg L^−1^ Se + 100 mg L^−1^ I (Figure 5l).

**Figure 6:**
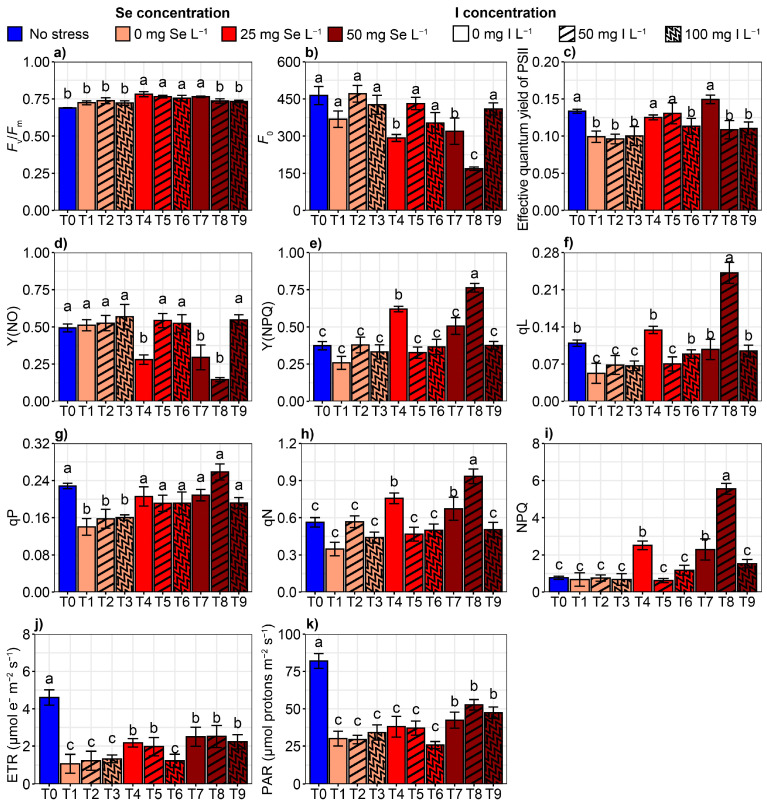
Effect of foliar application of Se and I, applied individually or in combination at different concentrations, on chlorophyll fluorescence parameters obtained using MINI-PAM in raspberry seedlings cv. ‘Heritage’ under non-stress and heat stress. (**a**) Maximum quantum efficiency of PSII (*F*_v_/*F*_m_), (**b**) minimum fluorescence (*F*_0_), (**c**) effective quantum yield of PSII [Y(II)], (**d**) quantum yield of non-regulated energy dissipation [Y(NO)], (**e**) quantum yield of regulated energy dissipation [Y(NPQ)], (**f**) photochemical quenching coefficient based on the lake model (qL), (**g**) photochemical quenching coefficient (qP), (**h**) non-photochemical quenching coefficient (qN), (**i**) non-photochemical quenching (NPQ), (**j**) electron transport rate (ETR), and (**k**) photosynthetically active radiation (PAR). The treatments included one non-stressed control (T0; 0 mg L^−1^ Se and 0 mg L^−1^ I) and nine stressed treatments. Among the stressed treatments, T1 (0 mg L^−1^ Se and 0 mg L^−1^ I) served as the stressed control, while T2 (0/50), T3 (0/100), T4 (25/0), T5 (25/50), T6 (25/100), T7 (50/0), T8 (50/50), and T9 (50/100) received different combinations of selenium and iodine. Values represent Se and I concentrations (mg L^−1^), respectively. Data are presented as the mean ± standard error. Different lowercase letters indicate significant differences among treatments according to the Scott–Knott test (*p* < 0.05).

Higher *F*_v_/*F*_m_ values were observed in seedlings treated with 25 mg L^−1^ Se, regardless of iodine concentration, and in seedlings treated with 50 mg L^−1^ Se alone. In contrast, the remaining treatments showed lower values (Figure 6a). For *F*_0_, higher values were recorded in the non-stressed control, the stressed control, the iodine-only treatments, and in treatments combining 25 mg L^−1^ Se with 50 or 100 mg L^−1^ I. In comparison, the lowest value was observed in seedlings treated with 50 mg L^−1^ Se + 50 mg L^−1^ I (Figure 6b). The effective quantum yield of PSII was higher in the non-stressed control and in seedlings treated with 25 mg L^−1^ Se alone or combined with 50 mg L^−1^ I, as well as in 50 mg L^−1^ Se alone, whereas lower values were observed in the remaining treatments (Figure 6c). For Y(NO), higher values were observed in the non-stressed control, the stressed control, the iodine-only treatments, and in seedlings treated with 25 mg L^−1^ Se combined with 50 or 100 mg L^−1^ I and 50 mg L^−1^ Se + 100 mg L^−1^ I. In contrast, lower values were recorded in seedlings treated with 25 mg L^−1^ Se alone and in treatments containing 50 mg L^−1^ Se combined with 0 or 50 mg L^−1^ I (Figure 6d).

Higher Y(NPQ), qL, qN, and NPQ values were observed in seedlings treated with 50 mg L^−1^ Se + 50 mg L^−1^ I, whereas lower values were generally recorded in the stressed control and iodine-only treatments (Figure 6e). For qP, higher values were observed in the non-stressed control and in seedlings treated with 25 mg L^−1^ Se and 50 mg L^−1^ Se, regardless of iodine concentration. In contrast, lower values were recorded in iodine-only treatments (Figure 6f). The highest ETR (Figure 6j) and PAR (Figure 6k) values were observed in the non-stressed control, whereas lower values were recorded in the stressed control, iodine-only treatments, and in seedlings treated with 25 mg L^−1^ Se + 100 mg L^−1^ I.

**Figure 7:**
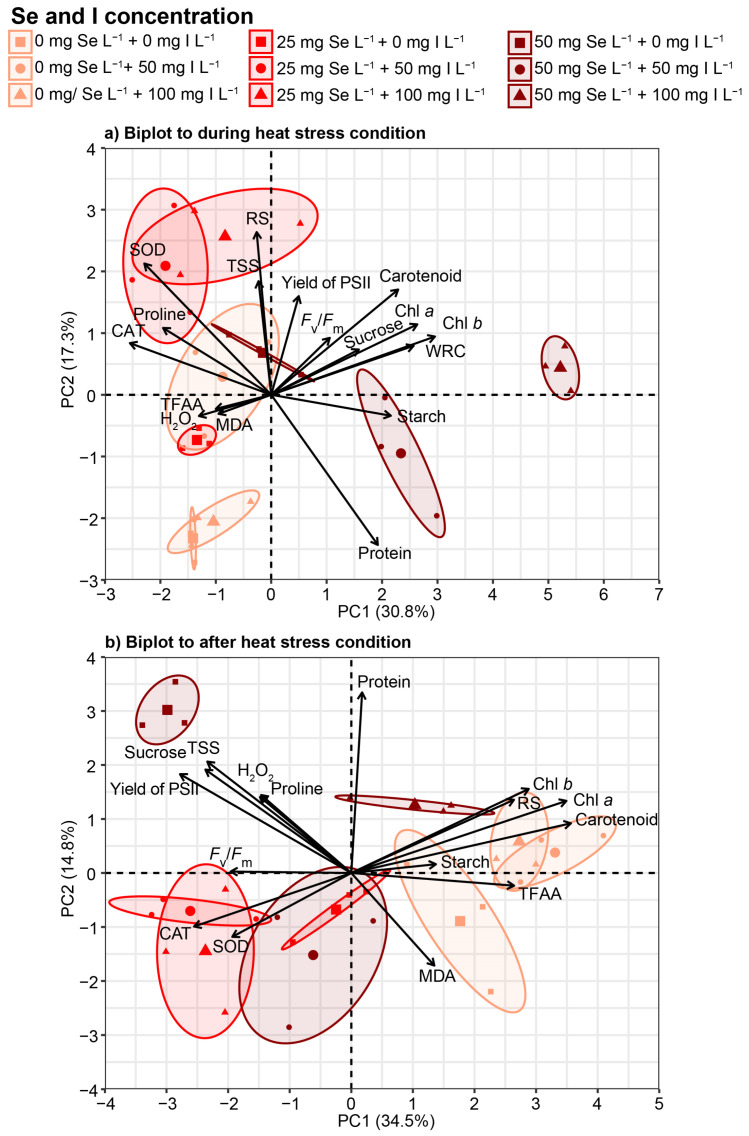
Principal component analysis (PCA) based on physiological, biochemical, and photochemical variables of raspberry seedlings cv. ‘Heritage’ was evaluated during (**a**) heat stress and (**b**) after heat stress following foliar application of selenium (Se) and iodine (I), applied individually or in combination at different concentrations. Variables included chlorophyll *a* (Chl *a*), chlorophyll *b* (Chl *b*), carotenoids, total soluble sugars (TSS), sucrose, starch, total free amino acids (TFAA), proline, protein, hydrogen peroxide (H_2_O_2_), malondialdehyde (MDA), catalase activity (CAT), superoxide dismutase activity (SOD), maximum quantum efficiency of PSII (*F*_v_/*F*_m_), effective quantum yield of PSII [Y(II)], and relative water content (RWC).

Under heat stress, principal component analysis (PCA) explained 48.1% of the total variance, with PC1 and PC2 accounting for 30.8% and 17.3% of the variation, respectively (Figure 7a). Seedlings treated with 25 mg L^−1^ Se combined with 50 or 100 mg L^−1^ I were positively associated with superoxide dismutase (SOD), catalase (CAT), proline, reducing sugars, and total soluble sugars, indicating a relationship with antioxidant activity and osmotic adjustment under heat stress (Figure 7a). In contrast, treatments containing 50 mg L^−1^ Se were more closely associated with photosynthetic variables, including chlorophyll *a*, chlorophyll *b*, carotenoids, effective quantum yield of PSII [Y(II)], *F*_v_/*F*_m_, sucrose, and starch content, suggesting improved maintenance of photosynthetic metabolism under heat stress (Figure 7a). The stressed control (0 mg L^−1^ Se + 0 mg L^−1^ I) was positioned on the negative side of PC1. It showed a lower association with antioxidant and osmotic adjustment variables than treatments receiving selenium, particularly those combined with iodine. In addition, seedlings treated with 25 mg L^−1^ Se alone were mainly associated with soluble protein content (Figure 7a).

After heat-stress recovery, the first two principal components explained 49.3% of the total variance, with PC1 and PC2 accounting for 34.5% and 14.8%, respectively (Figure 7b). Treatments containing 25 mg L^−1^ Se combined with iodine clustered in the positive region of PC1, where chlorophyll *a*, chlorophyll *b*, carotenoids, reducing sugars, and starch were also projected, suggesting an association with maintenance of photosynthetic pigments and carbohydrate metabolism after heat stress (Figure 7b). The stressed control occupied a distinct position in the ordination space and was separated from treatments associated with lipid peroxidation and amino acid accumulation. In contrast, the combination of 50 mg L^−1^ Se + 100 mg L^−1^ I was associated with malondialdehyde (MDA) and total free amino acids (TFAA), indicating a possible increase in metabolic imbalance under higher combined concentrations of Se and I (Figure 7b).

Overall, the PCA revealed distinct grouping patterns among treatments, indicating that intermediate Se concentrations combined with iodine were more strongly associated with antioxidant regulation, osmotic adjustment, maintenance of photosynthetic pigments, and preservation of photosynthetic performance under heat stress (Figure 7).

## 3. Discussion

Maintaining higher relative water content (RWC) in seedlings treated with combinations of 25 and 50 mg L^−1^ Se and iodine indicates that these elements help preserve plant water status under heat stress. Heat stress commonly disrupts membrane stability and increases transpiration rates, leading to cellular dehydration and impaired metabolic activity [17]. Selenium (Se) has been linked to maintaining membrane integrity, regulating stomatal conductance, and improving osmotic adjustment by modulating antioxidant metabolism and ROS detoxification [16,17]. In addition, iodine has recently been recognized as a functional element that modulates antioxidant signaling pathways and improves cellular homeostasis under abiotic stress [18].

The improved RWC observed in treatments combining Se with iodine (I) suggests that these treatments were associated with improved osmotic balance and maintenance of membrane function during heat exposure. However, because the underlying mechanisms were not directly investigated, the interactions between Se and I require further experimental validation [21]. These findings suggest that selenium and iodine may contribute through distinct physiological processes under heat stress. Based on previous studies, selenium has been associated with membrane stabilization and antioxidant regulation, whereas iodine has been linked to redox signaling and stress-responsive metabolic activation [17,18]. In contrast, seedlings treated with iodine alone showed lower RWC values, indicating that iodine alone may be insufficient to maintain water balance under severe thermal stress [22]. This result suggests that selenium plays an important role in protecting membrane function and maintaining water retention capacity under stress [25].

Although H_2_O_2_ concentrations varied significantly among treatments, MDA content did not. This apparent discrepancy indicates that ROS accumulation and lipid peroxidation were not proportional across treatments [26]. Hydrogen peroxide is a relatively stable ROS involved in stress signaling, whereas MDA is a downstream indicator of irreversible membrane lipid oxidation [27,28]. Therefore, increases in H_2_O_2_ may be associated with antioxidant and photoprotective responses before oxidative damage reaches the threshold required to trigger significant lipid peroxidation [29]. Additionally, the duration of heat stress may have been insufficient to induce severe membrane deterioration, particularly in seedlings with active antioxidant responses that maintained photosynthetic function [30]. Similar responses have been reported in plants subjected to moderate heat stress, where antioxidant systems efficiently limited membrane damage despite fluctuations in ROS accumulation [31,32].

The lower H_2_O_2_ accumulation observed in seedlings treated with iodine alone and in the combination of 25 mg L^−1^ Se + 100 mg L^−1^ I indicates improved ROS detoxification efficiency under heat stress. Selenium is known to regulate the activity of antioxidant enzymes, particularly superoxide dismutase (SOD), catalase (CAT), and ascorbate peroxidase (APX), thereby contributing to cellular redox homeostasis under stress [17]. Likewise, iodine has been associated with activation of antioxidant pathways and modulation of stress-responsive metabolism [18]. Thus, the combined application of Se and I was associated with differences in antioxidant responses among treatments; however, the present results do not confirm complementary or synergistic physiological mechanisms [16,17].

Maintaining higher chlorophyll and carotenoid levels in seedlings treated with combined Se and I applications demonstrates the protective effects of these elements on the photosynthetic apparatus. Heat stress accelerates chlorophyll degradation, destabilizes thylakoid membranes, and impairs photosystem II (PSII) activity due to excessive ROS production [33]. Selenium can protect chloroplast ultrastructure by stabilizing membrane systems and reducing oxidative damage, while iodine may help preserve photosynthetic pigments through antioxidant signaling and maintenance of redox balance [18,19,20]. The higher carotenoid concentrations observed in combined treatments are particularly relevant, as carotenoids play a central role in dissipating thermal energy and protecting against photooxidative stress [34]. The preservation of chlorophylls and carotenoids is physiologically relevant because these pigments are directly associated with light-harvesting efficiency and the dissipation of excess excitation energy [35]. Under heat stress, ROS overproduction accelerates pigment degradation and destabilizes PSII reaction centers, impairing electron transport and carbon assimilation [33]. Therefore, the higher pigment concentrations observed in seedlings treated with Se and I indicate improved protection of chloroplast structure and photosynthetic metabolism [23].

The accumulation of soluble sugars, sucrose, amino acids, and proline in several Se- and I-treated seedlings indicates activation of osmoprotective mechanisms under heat stress [18,36]. Osmolytes are essential for maintaining cell turgor, protecting proteins and membranes, and stabilizing enzymatic activity during stress [37]. Beyond osmotic adjustment, soluble sugars may also act as signaling molecules that regulate stress-responsive metabolism and ROS scavenging [23]. The higher proline accumulation in treatments with moderate Se and higher iodine concentrations suggests that these elements stimulate stress-acclimation pathways linked to osmoprotection and antioxidant defense [38,39].

The antioxidant enzyme responses further reinforce the role of Se and I in regulating cellular redox metabolism under heat stress [24,40]. The higher SOD activity observed in seedlings treated with 25 mg L^−1^ Se + 100 mg L^−1^ I indicates activation of the first enzymatic defense barrier against superoxide radicals [41,42]. Since SOD converts O_2_ into H_2_O_2_, coordinated CAT activity becomes essential to prevent excessive H_2_O_2_ accumulation [43]. The elevated CAT activity observed in treatments with moderate Se concentrations suggests efficient H_2_O_2_ detoxification and improved antioxidant homeostasis [44]. The coordinated regulation of SOD and CAT activities demonstrates that Se and I influence different steps in ROS detoxification pathways. While SOD dismutates superoxide radicals to H_2_O_2_, CAT subsequently detoxifies H_2_O_2_ to water and oxygen, thereby preventing oxidative propagation damage and maintaining cellular redox balance [43]. In contrast, the reduction in CAT activity at the highest combined Se and I concentrations may indicate an excessive element-induced redox imbalance or metabolic saturation, as reported under conditions of excessive Se accumulation [45].

Although total antioxidant capacity was not directly evaluated in the present study, the coordinated responses observed in SOD, CAT, H_2_O_2_, and lipid peroxidation suggest that the combined application of selenium and iodine may have improved the overall antioxidant status of raspberry seedlings under heat stress. However, because total antioxidant capacity was not directly measured, this inference remains indirect and should be confirmed in future studies using complementary biochemical approaches. The present study focused on physiological, biochemical, and enzymatic antioxidant responses to heat stress, and non-enzymatic antioxidant capacity was not assessed. Future studies integrating enzymatic antioxidant analyses with complementary assays, such as ABTS and FRAP, would provide a more comprehensive characterization of the antioxidant defense system underlying the physiological responses induced by selenium and iodine under heat stress.

Chlorophyll fluorescence responses indicate that Se and I applications help preserve PSII functionality under heat stress [23,46]. Seedlings treated with moderate Se combined with iodine maintain higher *F*_v_/*F*_m_ and effective quantum yield of PSII [Y(II)] values, indicating lower photoinhibition and improved photochemical efficiency [47]. Heat stress commonly damages the D1 protein of PSII and disrupts electron transport chains through ROS overproduction [48]. Selenium and iodine likely mitigate these effects by reducing oxidative damage and improving thylakoid membrane stability [49]. Moreover, higher qP and qL values indicate greater availability of open PSII reaction centers, suggesting improved photochemical utilization of absorbed light energy in treated seedlings [50]. These responses demonstrate that Se and I not only reduce oxidative damage but also help preserve the functional connectivity of PSII reaction centers under heat stress [51]. Maintaining electron transport efficiency is essential to prevent over-reduction of the photosynthetic apparatus and excessive ROS generation under high-temperature conditions [48].

In contrast, the increases in NPQ and qN observed in some treatments may reflect activation of photoprotective thermal dissipation mechanisms [52]. Although these mechanisms are essential for avoiding excessive excitation pressure under stress, excessively high NPQ values may also indicate increased energetic costs associated with stress acclimation [53]. Therefore, the balance between photochemical efficiency and thermal dissipation appears to be strongly influenced by the applied concentrations of Se and I.

The physiological responses observed throughout the experiment indicate that the interaction between selenium and iodine was concentration-dependent. Moderate combinations, particularly 25 mg L^−1^ Se combined with 50 or 100 mg L^−1^ I, consistently promoted greater antioxidant, osmotic, and photochemical adjustments than the isolated application of either element, suggesting beneficial interactions between selenium and iodine at specific concentration combinations under heat stress [16,17].

The PCA reinforced the physiological and biochemical responses observed throughout the experiment, demonstrating that intermediate Se concentrations, combined with iodine, were consistently associated with antioxidant metabolism, osmotic adjustment, and maintenance of photosynthetic performance under heat stress. These results suggest that treatments with moderate Se concentrations, combined with iodine, were associated with physiological and biochemical responses related to redox regulation, osmotic adjustment, and the maintenance of photosynthetic activity under heat stress. Previous studies have proposed that mechanisms such as ROS detoxification, osmoprotection, and photoprotection may contribute to these responses [24,32,38]. However, these mechanisms were not directly evaluated in the present study and therefore require further investigation in raspberry seedlings.

Although the present study demonstrates important physiological and biochemical responses to Se and I under heat stress, the underlying molecular mechanisms of their interaction were not directly evaluated. Future studies examining gene expression, antioxidant signaling pathways, ion accumulation, and chloroplast ultrastructure are needed to better elucidate the mechanistic basis of Se-I interactions in raspberry seedlings under heat stress. However, the lack of quantification of selenium and iodine in plant tissues is a major limitation, as it prevents direct confirmation of element uptake, translocation, and accumulation in response to the treatments.

Overall, the results indicate that the physiological responses to Se and I applications were strongly concentration-dependent. Moderate combinations were generally associated with antioxidant regulation, osmotic adjustment, and maintenance of photosynthetic function. In contrast, excessive concentrations, particularly under combined application, may exceed seedlings’ physiological buffering capacity, resulting in redox imbalance, increased energetic costs for stress acclimation, and partial impairment of antioxidant regulation. These findings reinforce the importance of optimizing Se and I management strategies to improve physiological acclimation and stress-associated metabolic adjustment in raspberry seedlings cultivated under subtropical and tropical conditions. Additionally, a non-stressed reference treatment should be included under different environmental conditions when interpreting comparisons across treatments, and future studies should include fully matched spray controls to isolate the effects of foliar application and environmental exposure.

## 4. Materials and Methods

### 4.1. Plant Material and Growth Conditions

The experiment was conducted in a greenhouse at the Department of Soil Science of the Federal University of Lavras (UFLA), in Lavras, Minas Gerais, Brazil (21°13′33.2″ S 44°58′43.3″ W; altitude 918 m). According to the Köppen climate classification, the regional climate is Cwb, a subtropical highland climate with dry winters and rainy summers [54]. During the experiment, the greenhouse temperature was maintained at 25 ± 2 °C during the day and 15 ± 2 °C at night.

Raspberry (*Rubus idaeus* L.) cv. ‘Heritage’ seedlings were produced by root-cutting propagation following the methodology described by Tadeu [55]. Seedlings averaged 26.0 ± 0.5 cm in height, had 10 to 12 fully expanded leaves, and exhibited a stem diameter of 3.0 ± 0.2 mm. Plants were grown in 2.5 L plastic bags filled with a commercial pine-bark-based substrate enriched with slow-release fertilizer (Basacot^®^, 9 months; COMPO ExpertGmbH, Münster, Germany) at 4 g per container. Irrigation was provided daily to maintain substrate moisture near field capacity, avoiding both water deficit and excessive moisture during the acclimatization phase.

### 4.2. Experimental Design and Treatment Application

The experiment was conducted in a completely randomized design (CRD) with four replicates, comprising ten treatments: one non-stressed control (T0; 0 mg L^−1^ Se and 0 mg L^−1^ I) and nine heat-stressed treatments. The stressed treatments, T1 (0 mg L^−1^ Se and 0 mg L^−1^ I), served as the stressed control, while the remaining treatments received foliar applications of selenium (Se; 0, 25, and 50 mg L^−1^) and iodine (I; 0, 50, and 100 mg L^−1^), applied individually or in combination. Thus, the experimental design included a non-stressed control, a stressed control, selenium-only treatments, iodine-only treatments, and combined Se + I treatments, allowing the individual and combined effects of both elements to be evaluated under heat stress conditions.

Each experimental unit consisted of three seedlings. For evaluations performed during the stress period, only the nine treatments subjected to combined heat and water stress were included in the statistical analyses. The non-stressed control (T0) was excluded because these plants were not exposed to the stress conditions, whereas the stressed control (T1; 0 mg L^−1^ Se and 0 mg L^−1^ I) was included together with the remaining stressed treatments. For post-stress evaluations, all ten treatments were analyzed, with the non-stressed control included as a physiological reference representing plants maintained under optimal growing conditions rather than as a treatment intended to evaluate the effects of selenium and iodine application (Table 1). The selected concentrations were based on preliminary assays and previous studies in different crop species that demonstrated beneficial effects of selenium and iodine on physiological acclimation and stress-associated responses while avoiding phytotoxic effects commonly associated with excessive concentrations of these elements [49,56,57].

Sodium selenate (Na_2_SeO_4_, reagent grade, Sigma-Aldrich, St. Louis, MO, USA) and potassium iodate (KIO_3_, reagent grade, Synth, Diadema, SP, Brazil) were used as Se and I sources, respectively. Mineral oil (0.20% *v*/*v*) was added to all sprayed treatments to improve foliar adherence and absorption. Foliar applications were performed inside a spraying chamber to avoid cross-contamination among treatments. Treatments were applied individually or in combination, as described in Table 1.

### 4.3. Heat Stress Conditions

One week after foliar application, seedlings were transferred to a growth chamber to impose heat stress. During the daytime period (12 h photoperiod), plants were exposed to 40 °C, 45% relative humidity, and photosynthetically active radiation of 260 μmol m^−2^ s^−1^. During the nighttime, the temperature was maintained at 18 °C and the relative humidity at 65%.

The seven-day interval between foliar application and stress imposition was established to allow adequate absorption, translocation, and metabolic action of selenium and iodine within plant tissues. During this interval, seedlings were visually monitored daily, and no visible symptoms of phytotoxicity or morphological alterations, including chlorosis, necrosis, leaf deformation, or reduced shoot vigor, were observed across treatments. No physiological or biochemical analyses were performed during this seven-day interval because the purpose of this period was to allow foliar absorption and physiological priming before heat stress was imposed. Therefore, all experimental evaluations were conducted during and after heat-stress exposure, in accordance with the experimental objectives. This approach was based on the priming concept, whereby these beneficial elements trigger physiological and biochemical adjustments that require time to become fully established before exposure to abiotic stress. Similar intervals have been used in previous studies evaluating the effects of selenium and iodine priming in broccoli seedlings subjected to cold stress and in coffee seedlings exposed to drought stress [49,57].

The daytime temperature of 40 °C was selected to induce acute heat stress conditions while avoiding irreversible damage to raspberry seedlings. Raspberry is a temperate fruit species with relatively low thermal tolerance, and temperatures above the optimum range for growth are known to impair photosynthesis, membrane stability, pigment preservation, and cellular redox balance. Previous studies have demonstrated that temperatures around 40 °C effectively trigger measurable physiological and biochemical heat stress responses while maintaining plant viability under controlled experimental conditions [15,25,30,33]. Therefore, 40 °C represents a severe but non-lethal heat-stress condition, allowing evaluation of protective mechanisms induced by selenium and iodine rather than irreversible tissue damage.

Heat stress was maintained for three consecutive days. Evaluations during heat stress were conducted after the first day of exposure. Post-stress evaluations were conducted immediately after the three-day heat-stress period, following plant removal from the growth chamber and the plant’s return to ambient greenhouse conditions.

### 4.4. Chlorophyll Fluorescence Analysis

After 1 day of heat-stress exposure, chlorophyll fluorescence was measured on six fully expanded leaves per treatment using a MINI-PAM-II Photosynthesis Yield Analyzer (Heinz Walz GmbH, Effeltrich, Germany). Measurements were conducted in the morning (08:00–10:00 h) under light-adapted conditions. The evaluated parameters included maximum quantum efficiency of photosystem II (*F*_v_/*F*_m_), minimum fluorescence (*F*_0_), effective quantum yield of photosystem II [Y(II)], regulated non-photochemical energy dissipation [Y(NPQ)], non-regulated non-photochemical energy dissipation [Y(NO)], photochemical quenching coefficients (qL and qP), non-photochemical quenching coefficient (qN), non-photochemical quenching (NPQ), electron transport rate (ETR), and photosynthetically active radiation (PAR).

### 4.5. Relative Water Content

Following chlorophyll fluorescence measurements, leaf samples were divided into two portions. One portion was used to determine relative water content (RWC) according to Barrs and Weatherley [58]. Leaf discs were weighed to obtain fresh weight (FW), hydrated in distilled water for 24 h in the dark to obtain turgid weight (TW), and subsequently dried in a forced-air oven at 65 °C until a constant weight was obtained to obtain dry weight (DW). Relative water content was calculated using the following equation:

$$RWC(\%)=\frac{FW-DW}{TW-DW}\times 100$$

The remaining leaf material was immediately frozen in liquid nitrogen and stored at −80 °C for subsequent biochemical analyses.

### 4.6. Biochemical Analyses

#### 4.6.1. Photosynthetic Pigments and Oxidative Stress Markers

Biochemical analyses included the quantification of chlorophyll *a*, chlorophyll *b*, carotenoids, hydrogen peroxide (H_2_O_2_), and malondialdehyde (MDA). For these analyses, ethanolic extraction was performed as described by López-Hidalgo et al. [59]. Briefly, 50 mg of frozen leaf tissue were subjected to sequential extraction using ethanol solutions of decreasing concentrations (100%, 80%, and 50%). The ethanolic extraction protocol was adopted for its efficiency in extracting multiple metabolites simultaneously and for its reduced interference from photosynthetic pigments during spectrophotometric analyses.

Chlorophyll *a*, chlorophyll *b*, and carotenoid contents were quantified spectrophotometrically from absorbance readings at 647, 623, and 450 nm, respectively. Hydrogen peroxide content was determined by the potassium iodide reaction following Alexieva et al. [60], with absorbance measured at 390 nm. Lipid peroxidation was estimated by quantifying malondialdehyde (MDA) using the thiobarbituric acid reaction described by Heath and Packer [61], with absorbance measured at 532 and 600 nm.

#### 4.6.2. Osmoprotectants, Carbohydrates, and Proteins

Reducing sugars were quantified using the dinitrosalicylic acid (DNS) method, as described by Miller [62]. Total soluble sugars and starch were determined by the anthrone method, as described by Chow and Landhäusser [63]. Sucrose was determined by alkaline extraction, followed by the anthrone reaction.

Free amino acids and proline contents were determined by the ninhydrin reaction method using spectrophotometric analysis at 550/570 and 520 nm, respectively. Protein was extracted from the pellet remaining after ethanolic extraction using a NaOH solution, and protein content was determined according to Bradford [64].

#### 4.6.3. Antioxidant Enzyme Activity

For antioxidant enzyme extraction, 0.20 g of fresh leaf tissue was macerated in liquid nitrogen and homogenized in an extraction buffer containing 100 mM potassium phosphate (pH 7.0), 0.1 mM EDTA, 1 mM dithiothreitol (DTT), 1 mM phenylmethylsulfonyl fluoride (PMSF), 1 mM ascorbic acid, and 1% (*w*/*v*) polyvinylpolypyrrolidone (PVPP), according to Azevedo et al. [65]. The homogenate was centrifuged at 13,000× *g* for 10 min at 4 °C, and the supernatant was used for enzymatic analyses.

Superoxide dismutase (SOD) activity was measured using the method of Beauchamp and Fridovich [66], which is based on inhibition of nitroblue tetrazolium (NBT) photoreduction at 560 nm. Catalase (CAT) activity was measured as described by Azevedo et al. [65] by monitoring H_2_O_2_ consumption at 240 nm.

### 4.7. Statistical Analysis

All statistical analyses were performed using the R statistical software version 4.5.0; R Foundation for Statistical Computing, Vienna, Austria) [67] using the packages stats, agricolae, corrplot, factoextra, FactoMineR, and Metrics [68,69,70,71]. Before analysis, the assumptions of analysis of variance (ANOVA), including normality, homogeneity of variances, additivity, and independence of residuals, were verified. When necessary, data were subjected to a square-root transformation (√x) to meet these assumptions before statistical analysis. For variables evaluated during the stress period, only the nine stressed treatments were analyzed using one-way ANOVA, with treatment considered as a single factor. The additional non-stressed control treatment was included in the experiment as a physiological reference. It was therefore excluded from these analyses because it was not subjected to stress conditions. For variables evaluated after the stress period, all 10 treatments were analyzed using one-way ANOVA, with treatment as the single factor. When significant, means were compared using the Scott–Knott test at *p* ≤ 0.05. Complete ANOVA summaries, including F-values and *p*-values, are provided in Supplementary Table S1.

Principal component analysis (PCA) was performed using the mean values of all physiological, biochemical, and photochemical variables evaluated in the experiment to investigate multivariate relationships among treatments under heat stress and after recovery, as well as among variables that showed significant differences in univariate analyses.

## 5. Conclusions

Combined foliar application of selenium and iodine modulated physiological, biochemical, and photochemical responses in raspberry seedlings under heat stress. Intermediate concentrations—particularly 25 mg L^−1^ Se combined with 50 or 100 mg L^−1^ I—were most consistently associated with maintenance of relative water content, antioxidant activity, osmotic adjustment, and photosynthetic performance. These findings suggest that Se and I supplementation may support physiological acclimation to heat stress. However, further studies assessing plant growth, survival, recovery, and tissue element accumulation are needed to determine whether these responses translate into improved whole-plant performance under heat stress.

## Figures and Tables

**Table 1:** Description of treatments used in the experiment.

Treatment	Se (mg L^−1^)	I (mg L^−1^)
T0 (non-stressed control)	0	0
T1 (stressed control)	0	0
T2	0	50
T3	0	100
T4	25	0
T5	25	50
T6	25	100
T7	50	0
T8	50	50
T9	50	100

The treatments included one non-stressed control (T0; 0 mg L^−1^ Se and 0 mg L^−1^ I) and nine water-stressed treatments. Among the stressed treatments, T1 (0 mg L^−1^ Se and 0 mg L^−1^ I) served as the stressed control, while T2 (0/50), T3 (0/100), T4 (25/0), T5 (25/50), T6 (25/100), T7 (50/0), T8 (50/50), and T9 (50/100) received different combinations of selenium and iodine. Values represent Se and I concentrations (mg L^−1^), respectively.

## Data Availability

The original contributions presented in this study are included in the article. Further inquiries can be directed to the corresponding author.

## Supplementary Materials

The following supporting information can be downloaded at: https://www.mdpi.com/article/10.3390/plants15142146/s1, Table S1. Summary of the analysis of variance (ANOVA) for all evaluated variables during the stress period. Table S2. Summary of the analysis of variance (ANOVA) for all evaluated variables after the stress period.
