# Supplementary material for: Combined Selenium and Iodine Application Modulates Antioxidant and Photosynthetic Responses in Raspberry Seedlings Under Heat Stress
Source: Plants (Basel). 2026 Jul 12;15(14):2146. doi: 10.3390/plants15142146 (PMC13414644; doi:10.3390/plants15142146)
Supplement: Supplementary file 1 [file plants-15-02146-s001.zip › plants-4401055-supplementary.pdf]

Supplementary Table S1. Summary of the analysis of variance (ANOVA) for all evaluated variables during the stress period.

Summary of the analysis of variance table for Water relative content$^{(sqrt)}$					
	**Df**	**Sum Square**	**Mean Square**	**F value**	**Pr(>F)**
Treatment	8	151.001	18.8751	37.023	0.0000
Residuals	18	9.177	0.5098		

Summary of the analysis of variance table for MDA content					
	**Df**	**Sum Square**	**Mean Square**	**F value**	**Pr(>F)**
Treatment	8	2248.6	281.07	0.9611	0.4946
Residuals	18	5263.9	292.44		

Summary of the analysis of variance table for H_2O_2 content					
	**Df**	**Sum Square**	**Mean Square**	**F value**	**Pr(>F)**
Treatment	8	2.56216	0.32027	6.4547	0.0005
Residuals	18	0.89312	0.04962		

Summary of the analysis of variance table for Fv/Fm values					
	**Df**	**Sum Square**	**Mean Square**	**F value**	**Pr(>F)**
Treatment	8	0.00512	0.00064	3.2013	0.0192
Residuals	18	0.0036	0.0002		

Summary of the analysis of variance table for F0					
	**Df**	**Sum Square**	**Mean Square**	**F value**	**Pr(>F)**
Treatment	8	3998.6	499.83	7.0992	0.0003
Residuals	18	1267.3	70.41		

Summary of the analysis of variance table for Effective quantum of yield of PSII					
	**Df**	**Sum Square**	**Mean Square**	**F value**	**Pr(>F)**
Treatment	8	0.06568	0.00821	17.18	0.0000
Residuals	18	0.0086	0.00048		

Summary of the analysis of variance table for Y(N0)					
	**Df**	**Sum Square**	**Mean Square**	**F value**	**Pr(>F)**
Treatment	8	0.15988	0.01999	2.9259	0.0279
Residuals	18	0.12295	0.00683		

Summary of the analysis of variance table for Y (NPQ)					
	**Df**	**Sum Square**	**Mean Square**	**F value**	**Pr(>F)**
Treatment	8	0.14894	0.01862	3.0686	0.0229
Residuals	18	0.10921	0.00607		

Summary of the analysis of variance table for qL					
	**Df**	**Sum Square**	**Mean Square**	**F value**	**Pr(>F)**
Treatment	8	0.01719	0.00215	3.1655	0.0201
Residuals	18	0.01222	0.00068		

Summary of the analysis of variance table for qP					
	**Df**	**Sum Square**	**Mean Square**	**F value**	**Pr(>F)**
Treatment	8	0.16357	0.02045	13.145	0.0000
Residuals	18	0.028	0.00156		

Summary of the analysis of variance table for qN					
	**Df**	**Sum Square**	**Mean Square**	**F value**	**Pr(>F)**
Treatment	8	0.52941	0.06618	5.8521	0.0009
Residuals	18	0.20355	0.01131		

Summary of the analysis of variance table for NPQ					
	**Df**	**Sum Square**	**Mean Square**	**F value**	**Pr(>F)**
Treatment	8	0.73369	0.09171	2.0222	0.1024
Residuals	18	0.81634	0.04535		

Summary of the analysis of variance table for $ETR^{(sqrt)}$					
	**Df**	**Sum Square**	**Mean Square**	**F value**	**Pr(>F)**
Treatment	8	1.0605	0.13256	2.1095	0.0899
Residuals	18	1.1311	0.06284		

Summary of the analysis of variance table for PAR					
	**Df**	**Sum Square**	**Mean Square**	**F value**	**Pr(>F)**
Treatment	8	1878.5	234.81	3.1732	0.0199
Residuals	18	1332	74		

Summary of the analysis of variance table for total soluble sugars					
	**Df**	**Sum Square**	**Mean Square**	**F value**	**Pr(>F)**
Treatment	8	12385	1548.13	4.0412	0.0066
Residuals	18	6895.6	383.09		

Summary of the analysis of variance table for Starch					
	**Df**	**Sum Square**	**Mean Square**	**F value**	**Pr(>F)**
Treatment	8	302.76	37.845	4.1721	0.0057
Residuals	18	163.28	9.071		

Summary of the analysis of variance table for total free amino acids					
	**Df**	**Sum Square**	**Mean Square**	**F value**	**Pr(>F)**
Treatment	8	320.28	40.035	13.342	0.0000
Residuals	18	54.01	3.001		

Summary of the analysis of variance table for chlorophyll *a* content					
	**Df**	**Sum Square**	**Mean Square**	**F value**	**Pr(>F)**
Treatment	8	1.35427	0.16928	28.527	0.0000
Residuals	18	0.10681	0.00593		

Summary of the analysis of variance table for chlorophyll *b* content					
	**Df**	**Sum Square**	**Mean Square**	**F value**	**Pr(>F)**
Treatment	8	0.47242	0.05905	19.765	0.0000
Residuals	18	0.05378	0.00299		

Summary of the analysis of variance table for total chlorophyll content					
	**Df**	**Sum Square**	**Mean Square**	**F value**	**Pr(>F)**
Treatment	8	0.45032	0.05629	36.16	0.0000
Residuals	18	0.02802	0.00156		

Summary of the analysis of variance table for carotenoids content					
	**Df**	**Sum Square**	**Mean Square**	**F value**	**Pr(>F)**
Treatment	8	1.03318	0.12915	16.194	0.0000
Residuals	18	0.14355	0.00798		

Summary of the analysis of variance table for proline					
	**Df**	**Sum Square**	**Mean Square**	**F value**	**Pr(>F)**
Treatment	8	3197114	399639	2.773	0.0345
Residuals	18	2594125	144118		

Summary of the analysis of variance table for protein					
	**Df**	**Sum Square**	**Mean Square**	**F value**	**Pr(>F)**
Treatment	8	19.7205	2.46506	20.866	0.0000
Residuals	18	2.1264	0.11814		

Summary of the analysis of variance table for sucrose					
	**Df**	**Sum Square**	**Mean Square**	**F value**	**Pr(>F)**
Treatment	8	4110.9	513.86	5.0396	0.0021
Residuals	18	1835.4	101.96		

Summary of the analysis of variance table for reducing sugars					
	**Df**	**Sum Square**	**Mean Square**	**F value**	**Pr(>F)**
Treatment	8	71390	8923.8	7.6715	0.0002
Residuals	18	20938	1163.2		

Summary of the analysis of variance table for catalase activity					
	**Df**	**Sum Square**	**Mean Square**	**F value**	**Pr(>F)**
Treatment	8	2.09388	0.26174	16.437	0.0000
Residuals	18	0.28662	0.01592		

Summary of the analysis of variance table for superoxide dismutase activity					
	**Df**	**Sum Square**	**Mean Square**	**F value**	**Pr(>F)**
Treatment	8	2439.09	304.89	49.818	0.0000
Residuals	18	110.16	6.12		

Df, degrees of freedom; Sum Square, sum of squares; Mean Square, mean square; F value, F statistic; Pr(>F), probability associated with the F-test (p-value). [sqrt:] *To meet the assumptions, the variable was transformed using the square root.*

Supplementary Table S2. Summary of the analysis of variance (ANOVA) for all evaluated variables after the stress period.

Summary of the analysis of variance table for MDA content					
	**Df**	**Sum Square**	**Mean Square**	**F value**	**Pr(>F)**
Treatment	9	4357	484.12	1.8475	0.1213
Residuals	20	5240.8	262.04		

Summary of the analysis of variance table for H_2O_2 content					
	**Df**	**Sum Square**	**Mean Square**	**F value**	**Pr(>F)**
Treatment	9	4.3782	0.48647	9.4082	0.0000
Residuals	20	1.0341	0.05171		

Summary of the analysis of variance table for Fv/Fm values					
	**Df**	**Sum Square**	**Mean Square**	**F value**	**Pr(>F)**
Treatment	9	0.019072	0.002119	3.7604	0.0065
Residuals	20	0.01127	0.000564		

Summary of the analysis of variance table for F0					
	**Df**	**Sum Square**	**Mean Square**	**F value**	**Pr(>F)**
Treatment	9	231057	25673	7.7402	0.0001
Residuals	20	66337	3316.8		

Summary of the analysis of variance table for Effective quantum of yield of PSII					
	**Df**	**Sum Square**	**Mean Square**	**F value**	**Pr(>F)**
Treatment	9	0.008237	0.000915	3.5777	0.0084
Residuals	20	0.005116	0.000256		

Summary of the analysis of variance table for Y(N0)					
	**Df**	**Sum Square**	**Mean Square**	**F value**	**Pr(>F)**
Treatment	9	0.58058	0.064509	8.0072	0.0001
Residuals	20	0.16113	0.008056		

Summary of the analysis of variance table for Y (NPQ)					
	**Df**	**Sum Square**	**Mean Square**	**F value**	**Pr(>F)**
Treatment	9	0.64697	0.071885	14.319	0.0000
Residuals	20	0.1004	0.00502		

Summary of the analysis of variance table for qL$^{(sqrt)}$					
	**Df**	**Sum Square**	**Mean Square**	**F value**	**Pr(>F)**
Treatment	9	0.15551	0.017279	10.415	0.0000
Residuals	20	0.03318	0.001659		

Summary of the analysis of variance table for qP					
	**Df**	**Sum Square**	**Mean Square**	**F value**	**Pr(>F)**
Treatment	9	0.033074	0.003675	4.5238	0.0024
Residuals	20	0.016247	0.000812		

Summary of the analysis of variance table for qN					
	**Df**	**Sum Square**	**Mean Square**	**F value**	**Pr(>F)**
Treatment	9	0.79132	0.087924	9.2552	0.0000
Residuals	20	0.19	0.0095		

Summary of the analysis of variance table for NPQ					
	**Df**	**Sum Square**	**Mean Square**	**F value**	**Pr(>F)**
Treatment	9	63.585	7.065	27.293	0.0000
Residuals	20	5.177	0.2589		

Summary of the analysis of variance table for ETR$^{(sqrt)}$					
	**Df**	**Sum Square**	**Mean Square**	**F value**	**Pr(>F)**
Treatment	9	3.2321	0.35912	4.3327	0.0030
Residuals	20	1.6577	0.08289		

Summary of the analysis of variance table for PAR					
	**Df**	**Sum Square**	**Mean Square**	**F value**	**Pr(>F)**
Treatment	9	7254.8	806.09	12.446	0.0000
Residuals	20	1295.3	64.77		

Summary of the analysis of variance table for total soluble sugars					
	**Df**	**Sum Square**	**Mean Square**	**F value**	**Pr(>F)**
Treatment	9	55151	6127.9	9.0111	0.0000
Residuals	20	13601	680		

Summary of the analysis of variance table for Starch					
	**Df**	**Sum Square**	**Mean Square**	**F value**	**Pr(>F)**
Treatment	9	443.7	49.3	7.7936	0.0001
Residuals	20	126.51	6.326		

Summary of the analysis of variance table for total free amino acids					
	**Df**	**Sum Square**	**Mean Square**	**F value**	**Pr(>F)**
Treatment	9	76.189	8.4655	7.6173	0.0001
Residuals	20	22.227	1.1113		

Summary of the analysis of variance table for chlorophyll *a* content					
	**Df**	**Sum Square**	**Mean Square**	**F value**	**Pr(>F)**
Treatment	9	0.77144	0.085715	7.3891	0.0001
Residuals	20	0.232	0.0116		

Summary of the analysis of variance table for chlorophyll *b* content					
	**Df**	**Sum Square**	**Mean Square**	**F value**	**Pr(>F)**
Treatment	9	0.34459	0.038288	6.0331	0.0004
Residuals	20	0.12693	0.006346		

Summary of the analysis of variance table for total chlorophyll content$^{(sqrt)}$					
	**Df**	**Sum Square**	**Mean Square**	**F value**	**Pr(>F)**
Treatment	9	0.279405	0.031045	8.5937	0.0000
Residuals	20	0.072251	0.003613		

Summary of the analysis of variance table for carotenoids content					
	**Df**	**Sum Square**	**Mean Square**	**F value**	**Pr(>F)**
Treatment	9	0.5718	0.063533	8.1054	0.0001
Residuals	20	0.15677	0.007838		

Summary of the analysis of variance table for proline					
	**Df**	**Sum Square**	**Mean Square**	**F value**	**Pr(>F)**
Treatment	9	3304899	367211	3.905	0.0054
Residuals	20	1880743	94037		

Summary of the analysis of variance table for protein					
	**Df**	**Sum Square**	**Mean Square**	**F value**	**Pr(>F)**
Treatment	9	12.8761	1.43068	5.489	0.0007
Residuals	20	5.2129	0.26065		

Summary of the analysis of variance table for sucrose					
	**Df**	**Sum Square**	**Mean Square**	**F value**	**Pr(>F)**
Treatment	9	4416.5	490.72	6.7516	0.0002
Residuals	20	1453.6	72.68		

Summary of the analysis of variance table for reducing sugars					
	**Df**	**Sum Square**	**Mean Square**	**F value**	**Pr(>F)**
Treatment	9	122388	13598.7	13.243	0.0000
Residuals	20	20538	1026.9		

Summary of the analysis of variance table for catalase activity					
	**Df**	**Sum Square**	**Mean Square**	**F value**	**Pr(>F)**
Treatment	9	0.100286	0.011143	13.646	0.0000
Residuals	20	0.016331	0.000817		

Summary of the analysis of variance table for superoxide dismutase activity					
	**Df**	**Sum Square**	**Mean Square**	**F value**	**Pr(>F)**
Treatment	9	336.73	37.415	15.163	0.0000
Residuals	20	49.35	2.468		

Df, degrees of freedom; Sum Square, sum of squares; Mean Square, mean square; F value, F statistic; Pr(>F), probability associated with the F-test (p-value). [sqrt:] *To meet the assumptions, the variable was transformed using the square root.*
